# Arthroscopic meniscal repair versus partial meniscectomy for middle-aged patients with meniscal tears and type 2 diabetes mellitus: A retrospective study on mid-to-long-term outcomes and prognostic factors

**DOI:** 10.1097/MD.0000000000047112

**Published:** 2026-02-13

**Authors:** Zhigang Zhou, Qiaoying Peng, Zheyuan Shen

**Affiliations:** a Department of Orthopedics, The First Affiliated Hospital of Huzhou Normal University, The First People’s Hospital of Huzhou, Huzhou City, Zhejiang Province, China.

**Keywords:** arthroscopy, functional recovery, logistic regression analysis, meniscal repair, meniscal tear, MRI follow-up, type 2 diabetes mellitus

## Abstract

The meniscus plays a critical role in maintaining knee joint stability, absorbing shock, and distributing load-bearing stress. Middle-aged individuals are prone to meniscal tears due to degenerative changes, while type 2 diabetes mellitus (T2DM), a prevalent chronic metabolic disorder, may impair cartilage healing and postoperative recovery, potentially compromising surgical outcomes. However, comparative evidence regarding arthroscopic meniscal repair versus partial meniscectomy in this specific population remains limited. This retrospective cohort study included 122 middle-aged patients with T2DM who underwent arthroscopic treatment for meniscal tears at our center between January 2023 and May 2024. Patients were divided into a repair group (n = 62) and a resection group (n = 60) based on the surgical procedure. Clinical outcomes within 12 months postoperatively were compared, including knee function (Lysholm and International Knee Documentation Committee scores), pain relief (Visual Analog Scale score), complication rate, and magnetic resonance imaging-based imaging findings. Multivariate logistic regression analysis was performed to identify independent predictors of functional recovery. Baseline characteristics were comparable between groups (all *P* > .05). At both 6 and 12 months postoperatively, the repair group showed significantly better Lysholm and International Knee Documentation Committee scores compared to the resection group (*P* < .001), along with consistently lower Visual Analog Scale scores (*P* < .01). Magnetic resonance imaging follow-up revealed a lower rate of cartilage degeneration in the repair group (Outerbridge grade ≥ 2: 16.1% vs 30.0%, *P* = .048), and the meniscal healing rate reached 85.5%. Complication rates were similar between groups (9.7% vs 11.7%, *P* = .71). Multivariate analysis identified surgical approach (repair: odds ratio [OR] = 1.92, *P* = .016), diabetes duration >10 years (OR = 1.78, *P* = .022), and preoperative glycated hemoglobin >7.5% (OR = 1.66, *P* = .031) as independent predictors of functional outcome. In middle-aged patients with T2DM and meniscal tears, arthroscopic meniscal repair offers superior outcomes in terms of functional recovery, pain relief, and cartilage preservation compared to partial meniscectomy, with comparable safety profiles. Patients with better glycemic control and shorter diabetes duration are more likely to benefit, supporting the preference for tissue-preserving strategies when feasible.

## 1. Introduction

The meniscus is one of the most critical fibrocartilaginous structures in the knee joint, playing a vital role in load transmission, shock absorption, stress dispersion, joint stabilization, and lubrication.^[1,2]^ Anatomical integrity of the meniscus is essential for maintaining long-term knee function and stability. Middle-aged individuals are particularly susceptible to meniscal tears due to progressive age-related degenerative changes, high activity levels, and cumulative mechanical stress.^[3,4]^ Clinical data have demonstrated a significantly increased prevalence of meniscal tears in individuals aged 40 to 60 years, with a considerable proportion requiring surgical intervention.^[5–7]^ The advent and widespread application of arthroscopy have made minimally invasive treatment of meniscal injuries feasible, with the 2 most common surgical approaches being meniscal repair and partial meniscectomy.^[8]^ Meniscal repair aims to preserve as much native tissue as possible, thereby maintaining joint biomechanics and delaying cartilage degeneration. In contrast, partial meniscectomy offers quicker symptomatic relief and shorter recovery time by removing torn fragments and smoothing the meniscal edges.^[9,10]^ Although there are clear indications for each procedure, clinical decision-making remains challenging in borderline or complex cases.

Type 2 diabetes mellitus (T2DM) is a highly prevalent chronic metabolic disease worldwide and is increasingly affecting younger populations. Its incidence among middle-aged individuals has risen steadily in recent years.^[11]^ Epidemiological data from China indicate that the prevalence of T2DM in individuals aged 35 to 59 years approaches 15%, suggesting a substantial overlap between patients with meniscal tears and those with diabetes.^[12,13]^ Numerous studies have shown that diabetes negatively affects the physiological metabolism and reparative capacity of knee cartilage and meniscal tissue through mechanisms including chronic inflammation induced by hyperglycemia, endothelial dysfunction, chondrocyte apoptosis, and oxidative stress.^[14,15]^ Furthermore, diabetic patients face higher risks during postoperative recovery, including infections, delayed wound healing, glycemic variability, and synovitis.^[16]^ Therefore, surgical decision-making for meniscal tears in diabetic patients must take into account both systemic metabolic conditions and local tissue repair potential. Although existing literature generally supports the long-term benefits of meniscal repair, there is a lack of systematic evaluation of its efficacy, safety, and prognostic factors in diabetic populations, especially among middle-aged patients.^[17,18]^ For this subgroup, optimal surgical selection is crucial for preserving quality of life, delaying joint degeneration, and reducing the risk of reoperation.

Most comparative studies between meniscal repair and meniscectomy have been conducted in general populations, where heterogeneity in metabolic background limits the generalizability of findings to diabetic patients.^[19,20]^ Some small-sample studies have suggested a potential association between diabetes and reduced meniscal healing rates, but causal links between surgical method and long-term outcomes have not been clearly established.^[21,22]^ Moreover, existing studies often lack mid-to-long-term follow-up data, robust imaging evidence, and comprehensive analyses incorporating key metabolic variables such as diabetes duration and glycated hemoglobin (HbA1c) levels. Therefore, conducting a clinical study specifically targeting middle-aged patients with T2DM to compare surgical outcomes in terms of functional recovery, pain relief, cartilage preservation, and complication rates is of high clinical relevance. Such research could help optimize surgical strategies and facilitate individualized treatment planning.

Given this context, the present study adopted a retrospective cohort design, enrolling 122 middle-aged patients with meniscal tears and concomitant T2DM who underwent either arthroscopic meniscal repair or partial meniscectomy at our center between January 2023 and May 2024. Patients were followed for 12 months postoperatively, and evaluated using multiple outcome measures including Lysholm scores, International Knee Documentation Committee (IKDC) scores, Visual Analog Scale (VAS) scores, and magnetic resonance imaging (MRI)-based assessments. Postoperative complications were recorded and analyzed. Furthermore, logistic regression modeling was applied to identify independent predictors of postoperative functional outcomes, focusing on variables such as surgical type, diabetes duration, and preoperative HbA1c levels. This dual approach (clinical and mechanistic) aims to elucidate differences in surgical efficacy between the 2 procedures.

The innovative aspects of this study are as follows: first, the study population specifically focuses on middle-aged individuals with T2DM, offering systematic comparative insights into mid-term outcomes of different surgical strategies within this unique subgroup: an area previously underexplored in the literature. Second, the outcome assessment is comprehensive, encompassing clinical scores, imaging follow-up, and complication profiles, thereby enhancing the external validity of the findings. Third, the use of a multivariate prognostic model allows for the identification of key clinical predictors of postoperative recovery, providing evidence-based guidance for surgical decision-making and perioperative management. The findings of this study are expected to inform future individualized treatment strategies for meniscal injuries in diabetic patients and contribute to improved long-term joint function and quality of life.

## 2. Methods

### 2.1. Study design and patient selection

This study was approved by the Ethics Committee of The First People’s Hospital of Huzhou. This was a single-center retrospective controlled study conducted to evaluate the impact of different surgical procedures on postoperative outcomes in middle-aged patients with meniscal tears complicated by T2DM. A total of 122 patients who underwent arthroscopic knee surgery in our orthopedic department between January 2023 and May 2024 were enrolled. All patients had a confirmed diagnosis of unilateral meniscal tear by MRI and a documented history of T2DM.

Eligible patients were aged between 40 and 60 years, had a clearly defined meniscal tear pattern on MRI, maintained stable glycemic control without acute diabetic complications, showed no radiographic evidence of advanced osteoarthritis (Kellgren–Lawrence grade ≤ II), and were capable of adhering to follow-up visits and rehabilitation protocols. Patients were excluded if they required concurrent ligament reconstruction, had a history of previous knee surgery, or suffered from systemic rheumatic diseases, active infections, malignancies, or severe cardiopulmonary disorders.

Because this was a retrospective consecutive-case study, no a priori sample size calculation was performed. All eligible patients treated at our institution during the study period were included. A post hoc power analysis was conducted using the observed between-group difference in the 12-month IKDC score, which demonstrated a statistical power exceeding 0.80, indicating that the available sample size was sufficient to detect meaningful differences between the 2 surgical procedures

Based on the actual intraoperative procedures performed, patients were classified into 2 groups: the repair group (n = 62), who underwent arthroscopic meniscal suture repair, and the resection group (n = 60), who underwent partial meniscectomy.

### 2.2. Baseline data collection

Preoperative demographic and clinical data were extracted from the electronic medical records by 2 independent investigators. Collected variables included age, sex, height, weight, body mass index, systolic and diastolic blood pressure, resting heart rate, smoking history, duration of diabetes, HbA1c level, and type of meniscal tear. The tear morphology was classified as longitudinal, radial, horizontal, or complex based on a combination of preoperative MRI findings and intraoperative arthroscopic evaluation. All data were independently entered by 2 researchers and cross-verified for consistency.

### 2.3. Surgical procedures and postoperative management

All procedures were performed by the same experienced arthroscopy team. In the repair group, meniscal suturing was conducted using an all-inside, inside-out, or outside-in technique, selected according to the specific morphology and location of the tear, with the goal of preserving maximal tissue continuity. In the resection group, partial meniscectomy was performed to remove unstable fragments while preserving the load-bearing zones. Postoperatively, all patients received standardized analgesic, anti-inflammatory, antithrombotic, and glycemic control regimens. A stepwise rehabilitation protocol was initiated on the 1st postoperative day, gradually advancing to weight-bearing ambulation, with scheduled follow-up visits for clinical and imaging assessments.

### 2.4. Clinical outcome measures

Functional outcomes were assessed using the Lysholm Knee Scoring Scale and the IKDC subjective form at 6 and 12 months postoperatively. Pain was evaluated using the VAS at 3, 6, and 12 months. At the 12-month follow-up, MRI examinations were conducted to assess cartilage degeneration based on the Outerbridge classification and to evaluate meniscal healing status. Postoperative complications, including joint stiffness, synovial reactions, and glycemic fluctuations, were systematically recorded by the clinical care team. All imaging evaluations were independently reviewed by 2 experienced musculoskeletal radiologists blinded to the clinical data.

### 2.5. Statistical analysis

All statistical analyses were performed using SPSS version 25.0 (IBM Corp., Armonk). Continuous variables were expressed as mean ± standard deviation and compared between groups using the independent-samples *t* test. Categorical variables were expressed as frequencies and percentages and analyzed using the *χ*² test or Fisher exact test, as appropriate. Multivariate logistic regression analysis was conducted to identify independent predictors of favorable functional recovery, defined as an IKDC score ≥ 80 at 12 months postoperatively. Covariates entered into the regression model included surgical procedure, duration of diabetes, preoperative HbA1c level, age, sex, and tear type. Odds ratios (ORs) with 95% confidence intervals (CIs) were reported, and a 2-sided *P* value < .05 was considered statistically significant.

## 3. Result

### 3.1. Comparison of baseline characteristics

A total of 122 middle-aged patients with meniscal tears and T2DM were included in this study, with 62 patients in the repair group and 60 in the partial meniscectomy group. The 2 groups were generally balanced in terms of preoperative demographic and clinical characteristics.

The mean age was 49.2 ± 6.1 years in the repair group and 48.7 ± 6.4 years in the resection group (*t* = 0.495, *P* = .62), and the proportion of male patients was 58.1% versus 55.0%, respectively (*χ*² = 0.152, *P* = .73), with no statistically significant differences. Regarding body habitus, no significant differences were observed between the groups in height (168.7 ± 7.5 cm vs 167.9 ± 6.9 cm, *t* = 0.773, *P* = .44), weight (71.5 ± 9.2 kg vs 70.8 ± 8.7 kg, *t* = 0.551, *P* = .58), or body mass index (25.1 ± 2.6 vs 25.0 ± 2.8 kg/m², *t* = 0.241, *P* = .81).

Vital signs were also comparable between groups. The repair group had a mean systolic blood pressure of 132.5 ± 13.2 mm Hg, diastolic blood pressure of 81.4 ± 9.1 mm Hg, and resting heart rate of 75.2 ± 6.8 beats/min; while the corresponding values in the resection group were 131.8 ± 12.9 mm Hg, 80.7 ± 9.5 mm Hg, and 74.7 ± 7.3 beats/min, respectively (all *P* > .05).

Diabetes-related parameters showed no significant intergroup differences. The mean duration of diabetes was 8.3 ± 3.6 years in the repair group and 8.0 ± 3.9 years in the resection group (*t* = 0.582, *P* = .56); preoperative HbA1c levels were 7.4 ± 0.7% and 7.3 ± 0.7%, respectively (*t* = 1.056, *P* = .29). The proportion of patients with a history of smoking was similar between the 2 groups (29.0% vs 28.3%, *χ*² = 0.009, *P* = .92).

Regarding the types of meniscal tear, the distribution of longitudinal, radial, horizontal, and complex tear patterns did not significantly differ between the 2 groups (*χ*² = 2.449, *P* = .48). In summary, the baseline characteristics were well matched between groups, providing a reliable foundation for subsequent outcome comparisons. Detailed baseline data are presented in Table 1.

**Table 1:** Baseline characteristics of patients in the repair and resection groups.

Variable	Repair group (n = 62)	Resection group (n = 60)	*χ*²/*t* value	*P*-value
Age (yr)	49.2 ± 6.1	48.7 ± 6.4	*t* = 0.495	.62
Male [n, %]	36 (58.1%)	33 (55.0%)	*χ*² = 0.152	.73
Height (cm)	168.7 ± 7.5	167.9 ± 6.9	*t* = 0.773	.44
Weight (kg)	71.5 ± 9.2	70.8 ± 8.7	*t* = 0.551	.58
BMI (kg/m²)	25.1 ± 2.6	25.0 ± 2.8	*t* = 0.241	.81
Systolic BP (mm Hg)	132.5 ± 13.2	131.8 ± 12.9	*t* = 0.415	.68
Diastolic BP (mm Hg)	81.4 ± 9.1	80.7 ± 9.5	*t* = 0.524	.60
Resting heart rate (bpm)	75.2 ± 6.8	74.7 ± 7.3	*t* = 0.458	.65
Smoking history [n, %]	18 (29.0%)	17 (28.3%)	*χ*² = 0.009	.92
Duration of diabetes (yr)	8.3 ± 3.6	8.0 ± 3.9	*t* = 0.582	.56
HbA1c (%)	7.4 ± 0.7	7.3 ± 0.7	*t* = 1.056	.29
Meniscal tear type [n, %]			*χ*² = 2.449	.48
Longitudinal	21 (33.9%)	20 (33.3%)		
Radial	13 (21.0%)	14 (23.3%)		
Horizontal	15 (24.2%)	13 (21.7%)		
Complex	13 (21.0%)	13 (21.7%)		

BMI = body mass index, HbA1c = glycated hemoglobin.

### 3.2. Postoperative knee function recovery

During postoperative follow-up, knee joint function was assessed using the Lysholm score and the IKDC subjective score. The results demonstrated that the repair group achieved significantly better functional recovery than the partial meniscectomy group.

At 6 months postoperatively, the Lysholm score in the repair group was 83.1 ± 7.5, compared to 76.3 ± 8.6 in the resection group (*t* = 5.00, *P* < .001). Similarly, the IKDC score was 79.3 ± 7.8 in the repair group versus 72.1 ± 9.4 in the resection group (*t* = 4.66, *P* < .001), both showing statistically significant differences.

**Figure 1.**
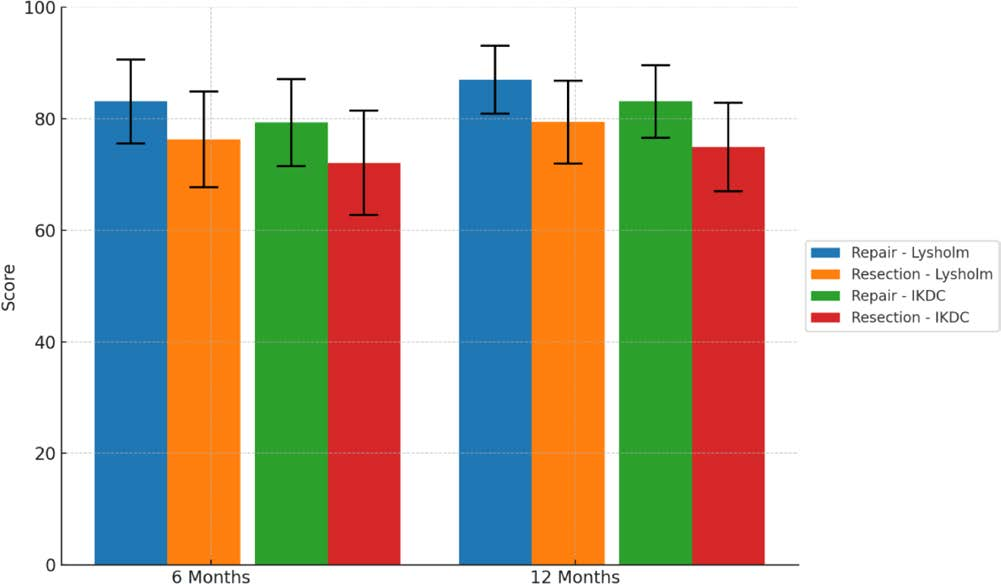
Postoperative functional scores at 6 and 12 months.

By 12 months postoperatively, the functional outcomes continued to improve. The Lysholm score further increased to 87.0 ± 6.1 in the repair group, while the resection group reached only 79.4 ± 7.4 (*t* = 6.11, *P* < .001). The IKDC scores at 12 months were 83.1 ± 6.5 and 74.9 ± 7.9, respectively (*t* = 6.05, *P* < .001), with the intergroup difference widening over time. These results are presented in Figure 1 and Table 2.

**Table 2:** Comparison of postoperative rehabilitation time among different groups.

Group	Lysholm score (6 mo)	Lysholm score (12 mo)	IKDC score (6 mo)	IKDC score (12 mo)
Repair	83.1 ± 7.5	87.0 ± 6.1	79.3 ± 7.8	83.1 ± 6.5
Resection	76.3 ± 8.6	79.4 ± 7.4	72.1 ± 9.4	74.9 ± 7.9
*P* value	<.001	<.001	<.001	<.001
*t* value	5.00	6.11	4.66	6.05

IKDC = International Knee Documentation Committee.

Both groups demonstrated a time-dependent improvement in functional scores; however, the repair group showed a significantly greater magnitude and faster pace of recovery. These findings suggest that arthroscopic meniscal repair offers superior long-term functional benefits over partial meniscectomy in middle-aged patients with T2DM.

### 3.3. Pain relief outcomes

Postoperative pain levels were assessed using the VAS, with scores ranging from 0 to 10, where higher values indicate greater pain severity. The results showed that both groups experienced substantial pain relief following surgery; however, the repair group exhibited superior analgesic effects during mid- to long-term follow-up.

**Figure 2.**
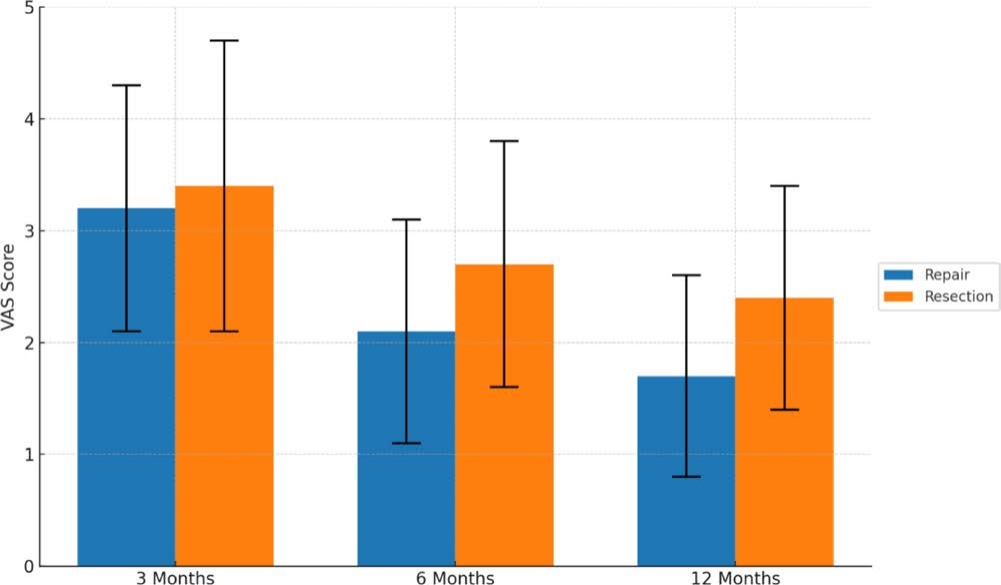
Postoperative recovery time.

At 3 months postoperatively, VAS scores were comparable between the 2 groups: 3.2 ± 1.1 in the repair group and 3.4 ± 1.3 in the resection group (*t* = 1.062, *P* = .29), indicating similar short-term pain control. By 6 months, the VAS score in the repair group had decreased to 2.1 ± 1.0, which was significantly lower than the 2.7 ± 1.1 observed in the resection group (*t* = 2.895, *P* = .005). At the 12-month follow-up, the difference widened further, with the repair group reporting a score of only 1.7 ± 0.9 compared to 2.4 ± 1.0 in the resection group (*t* = 3.971, *P* < .001). These results are illustrated in Figure 2 and detailed in Table 3.

**Table 3:** Comparison of VAS scores between the 2 groups at different time points.

Group	VAS score (3 mo)	VAS score (6 mo)	VAS score (12 mo)
Repair	3.2 ± 1.1	2.1 ± 1.0	1.7 ± 0.9
Resection	3.4 ± 1.3	2.7 ± 1.1	2.4 ± 1.0
*t* value	1.062	2.895	3.971
*P* value	.29	.005	<.001

VAS = Visual Analog Scale.

Collectively, the data suggest that although both surgical approaches provide comparable short-term pain relief, arthroscopic meniscal repair offers significantly better mid- to long-term pain control. This advantage may be attributed to preserved meniscal integrity and improved biomechanical stability of the knee joint following repair.

### 3.4. Incidence of postoperative complications

Postoperative complications occurring within 12 months after surgery were retrospectively analyzed. In the repair group, complications were observed in 6 patients (9.7%, 6/62), while 7 patients in the resection group experienced complications (11.7%, 7/60). The difference between groups was not statistically significant (*χ*² = 0.139, *P* = .71), suggesting comparable overall postoperative safety between the 2 surgical approaches (see Table 4).

**Table 4:** Comparison of postoperative complications between groups.

Group	Total complications [n, %]	Joint stiffness	Synovitis	Glycemic fluctuation	*χ*² value	*P*-value
Repair (n = 62)	6 (9.7%)	3	2	1	0.139	.71
Resection (n = 60)	7 (11.7%)	3	2	2		

Regarding the specific types of complications, joint stiffness was the most frequently reported adverse event, with 3 cases in each group. This was followed by postoperative synovial reactions, which occurred in 2 patients in both the repair and resection groups. Transient postoperative blood glucose fluctuations were reported in 1 case in the repair group and 2 cases in the resection group. All complications were effectively managed with conservative treatment, and no patient required revision surgery due to postoperative adverse events.

These findings indicate that arthroscopic meniscal repair does not significantly increase the risk of postoperative complications in middle-aged patients with T2DM, despite its tissue-preserving nature. This supports the clinical feasibility of meniscal repair as a safe and effective treatment option in this specific population.

### 3.5. Postoperative radiological follow-up

At 12 months postoperatively, all patients underwent MRI evaluation to assess cartilage status and meniscal healing. The findings demonstrated that patients in the repair group exhibited milder cartilage degeneration, indicating superior preservation of joint structure. Specifically, according to the Outerbridge grading system, the incidence of cartilage degeneration grade ≥2 was significantly lower in the repair group (16.1%, 10/62) compared to the resection group (30.0%, 18/60), with a statistically significant difference (*χ*² = 3.912, *P* = .048). This suggests that preservation of the meniscal structure may play a protective role in delaying the progression of cartilage degeneration.

In terms of meniscal healing, follow-up MRI in the repair group revealed a healing rate of 85.5% (53/62), with the majority showing continuous low-signal intensity and smooth articular surfaces, indicating satisfactory structural integrity. In contrast, due to partial removal of the meniscus, healing status in the resection group could not be evaluated, and therefore no direct comparison was possible.

Collectively, these radiological findings provide further evidence supporting the benefits of arthroscopic meniscal repair in protecting articular cartilage and promoting tissue healing. This advantage is particularly relevant for middle-aged patients with T2DM who require long-term joint preservation and functional stability.

### 3.6. Analysis of prognostic factors influencing postoperative outcomes

To identify key factors associated with postoperative functional recovery, a multivariate logistic regression analysis was performed on relevant clinical variables. Good recovery was defined as an IKDC score ≥80 at 12 months postoperatively. Covariates included in the model were surgical method, duration of diabetes, preoperative HbA1c level, sex, age, and tear type.

The regression results indicated that the type of surgical procedure was a significant protective factor. Compared with partial meniscectomy, arthroscopic meniscal repair was significantly associated with a higher likelihood of good postoperative functional recovery (OR = 1.92, 95% CI: 1.13–3.27, *P* = .016). In addition, a diabetes duration exceeding 10 years was identified as a negative prognostic factor (OR = 1.78, 95% CI: 1.09–2.91, *P* = .022), and a preoperative HbA1c level > 7.5% was also significantly associated with poorer functional outcomes (OR = 1.66, 95% CI: 1.05–2.63, *P* = .031).

**Figure 3.**
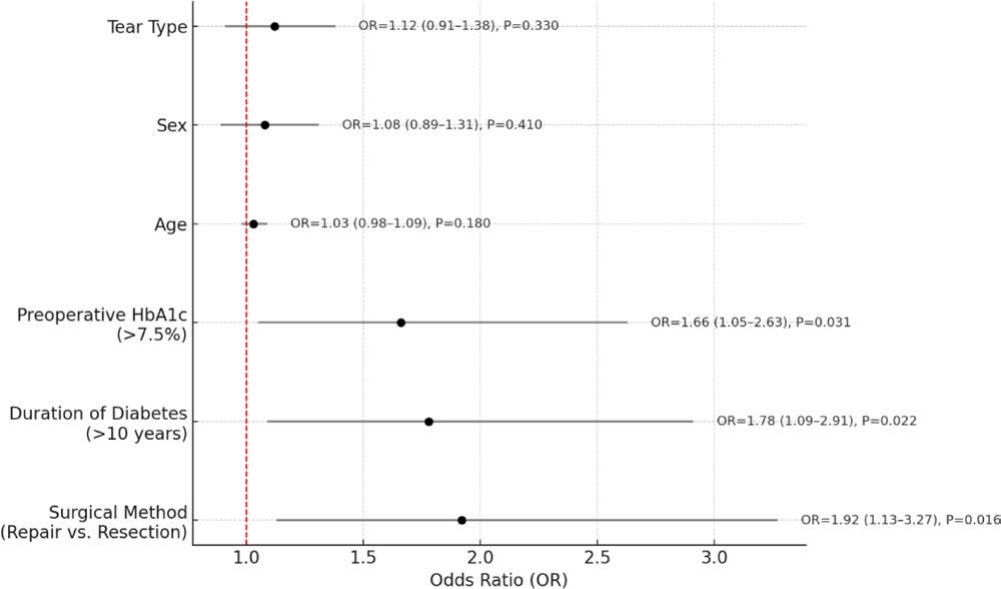
Multivariate logistic regression: factors influencing functional recovery.

Other variables, including sex (*P* = .41), age (*P* = .18), and meniscal tear type (*P* = .33), did not show significant associations in the multivariate model, suggesting limited predictive value for postoperative functional outcomes in this cohort. The detailed results are illustrated in Figure 3.

## 4. Discussion

The meniscus plays a critical role in maintaining knee joint stability, absorbing shock, and distributing mechanical loads. Its structural integrity is essential for preserving long-term joint function.^[23]^ Middle-aged individuals, due to the onset of physiological degeneration, are particularly prone to meniscal tears.^[24]^ Currently, arthroscopic meniscal repair and partial meniscectomy are the primary surgical interventions, and the choice between them is influenced by tear type, patient age, activity level, and comorbidities. Diabetes mellitus, a common chronic metabolic disorder, not only impairs the regenerative potential of meniscal and cartilaginous tissues but may also interfere with postoperative recovery. Although existing studies have reported superior functional outcomes with meniscal repair compared to meniscectomy, evidence remains limited in the specific population of patients with diabetes.^[25,26]^

This study investigated 122 middle-aged patients with T2DM who underwent either arthroscopic meniscal repair or partial meniscectomy. The clinical outcomes at 12 months postoperatively, including knee function, pain relief, complication rates, and MRI-based structural prognosis, were systematically compared. The results demonstrated that meniscal repair yielded significantly better mid-term outcomes in multiple domains without increasing the risk of complications, reinforcing the therapeutic value of tissue-preserving approaches in diabetic patients.

Consistent with prior research, we observed significantly higher Lysholm and IKDC scores at both 6 and 12 months postoperatively in the repair group compared to the meniscectomy group, indicating enhanced functional recovery.^[27,28]^ Previous literature has also highlighted the importance of preserving meniscal tissue in maintaining joint biomechanics and delaying the progression of cartilage degeneration and osteoarthritis.^[29]^ Nonetheless, the suitability of repair in diabetic patients has remained controversial due to concerns about impaired collagen synthesis, chronic inflammation, and microvascular pathology. Our findings suggest that diabetes, when well-controlled, should not be considered a contraindication for meniscal repair, and favorable outcomes can still be achieved.

In terms of pain relief, although VAS scores were comparable between groups at 3 months postoperatively, the repair group showed significantly lower pain levels at both 6 and 12 months. This difference may be attributed to the preservation of meniscal integrity, leading to more even load distribution and reduced mechanical irritation. While partial meniscectomy provides rapid symptomatic relief, long-term alterations in joint loading may contribute to chronic pain and functional decline.

MRI follow-up at 12 months further substantiated the advantages of meniscal repair. Patients in the repair group had significantly fewer cases of grade ≥2 cartilage degeneration based on the Outerbridge classification, suggesting superior joint protection. Moreover, the meniscal healing rate reached 85.5% in the repair group, with most cases showing continuous low-signal intensity and smooth articular surfaces, indicating stable repair and favorable tissue remodeling. These findings are particularly meaningful given the impaired cartilage repair potential in diabetic patients, emphasizing the protective effect of meniscal preservation in this population.

Additionally, no significant difference was observed in the incidence of postoperative complications between the 2 groups, with the most common events being joint stiffness and synovitis, all of which were successfully managed conservatively. Importantly, no cases of postoperative infection were reported, likely due to strict perioperative glycemic control and standardized management protocols. This supports the safety profile of meniscal repair in diabetic individuals when appropriate surgical and metabolic strategies are applied.^[30]^

Multivariate logistic regression analysis identified surgical method, duration of diabetes, and preoperative HbA1c level as significant predictors of postoperative outcomes. Specifically, undergoing meniscal repair significantly increased the likelihood of achieving good IKDC scores, while a diabetes duration >10 years and preoperative HbA1c >7.5% were associated with worse functional recovery. These findings highlight the importance of comprehensive metabolic assessment and optimization before surgery, and favor the selection of tissue-preserving techniques when feasible.

The key strengths of this study include: a focus on a high-risk and understudied population (middle-aged patients with T2DM) thus addressing a clinical knowledge gap; the use of multiple outcome measures including functional scores, pain scales, and MRI findings to ensure robust and comprehensive assessment; and the development of a multivariate regression model to identify prognostic factors, providing practical guidance for personalized treatment planning. Compared to previous studies which have largely focused on general populations or short-term outcomes, our design offers greater clinical relevance and applicability.

However, several limitations should be acknowledged. First, this was a single-center retrospective study, and selection bias could not be fully eliminated despite careful inclusion criteria. Second, the follow-up period was limited to 12 months, which may be insufficient to evaluate long-term outcomes such as osteoarthritis progression. Third, surgical approach was determined intraoperatively rather than through randomized allocation, which may introduce confounding. Future multicenter, long-term prospective randomized controlled trials are warranted to validate our findings and refine patient selection criteria for different surgical approaches. Another important limitation is that postoperative rehabilitation adherence and glycemic control during follow-up were not systematically captured in the clinical records. Both factors may significantly influence tissue healing and functional recovery, particularly in patients with T2DM. Although preoperative HbA1c was included as a predictor in the multivariate model, dynamic glycemic control over the 12-month recovery period could not be evaluated. Future prospective studies with standardized rehabilitation monitoring and longitudinal metabolic assessment are needed to better quantify their impact.

In conclusion, our study suggests that arthroscopic meniscal repair provides superior functional recovery, better pain control, and improved cartilage protection compared to partial meniscectomy in middle-aged patients with T2DM, without increasing the risk of complications. For patients with well-controlled metabolic status and appropriate tear patterns, meniscal repair should be the preferred option to optimize clinical and structural outcomes.

## 5. Conclusion

This study systematically compared the 12-month postoperative outcomes of arthroscopic meniscal repair versus partial meniscectomy in middle-aged patients with meniscal tears and T2DM. The results demonstrated that meniscal repair significantly outperformed partial meniscectomy in terms of functional recovery, pain relief, and cartilage protection, without increasing the incidence of postoperative complications. MRI follow-up further confirmed the structural benefits of meniscal preservation, including delayed cartilage degeneration and enhanced meniscal healing. Multivariate logistic regression identified surgical method, diabetes duration, and preoperative HbA1c level as independent predictors of functional outcomes, underscoring the importance of metabolic control and surgical decision-making in optimizing recovery. This study fills an evidence gap in the management of meniscal injuries in diabetic patients and highlights the necessity of prioritizing tissue-preserving strategies in this specific population. Future studies incorporating long-term follow-up and multicenter validation are warranted to refine individualized treatment strategies and improve long-term outcomes and quality of life for this high-risk group.

## Author contributions

**Conceptualization:** Zhigang Zhou, Qiaoying Peng, Zheyuan Shen.

**Data curation:** Zhigang Zhou, Qiaoying Peng, Zheyuan Shen.

**Formal analysis:** Zhigang Zhou, Qiaoying Peng, Zheyuan Shen.

**Funding acquisition:** Zhigang Zhou, Qiaoying Peng, Zheyuan Shen.

**Investigation:** Zheyuan Shen.

**Writing – original draft:** Zhigang Zhou, Qiaoying Peng, Zheyuan Shen.

**Writing – review & editing:** Zhigang Zhou, Zheyuan Shen.
